# Sleep in Bipolar Disorder: A Systematic Review and Meta-Analysis of Case-Control Drug-Free Patients

**DOI:** 10.3390/brainsci16070742

**Published:** 2026-07-13

**Authors:** Giuseppe Barbato, Barbara Tondi, Dario Morra

**Affiliations:** Dipartimento di Psicologia, Università della Campania “Luigi Vanvitelli”, 81100 Caserta, Italy; giuseppe.barbato@unicampania.it (G.B.); barbara.tondi.00@gmail.com (B.T.)

**Keywords:** bipolar disorder, mania, depression, sleep, REM sleep, delta sleep, meta-analysis, REM density, arousal

## Abstract

**Highlights:**

**What are the main findings?**

- Drug-free bipolar disorder is associated with reduced total sleep time and sleep efficiency in both depressive and manic phases.
- Bipolar depression shows reduced slow-wave sleep, whereas mania shows reduced REM latency and increased REM density.

**What are the implications of the main findings?**

- Sleep architecture shows phase-specific alterations in bipolar disorder, involving differential disruption of NREM slow-wave activity and REM regulation.
- Pharmacological treatment is associated with attenuation of several sleep abnormalities, consistent with a state-dependent pattern of bipolar polysomnographic alterations.

**Abstract:**

**Background**: A systematic review and meta-analysis of polysomnographic sleep parameters in bipolar disorder was conducted using articles identified through searches of major databases from inception to 5 May 2026. **Methods**: One hundred and seven studies were identified in the systematic review. Forty-three case-control studies with 670 bipolar patients, 520 healthy controls and 280 patients with unipolar depression were eligible for the meta-analyses. Total sleep time, sleep onset latency, sleep efficiency, wake after sleep onset, REM time and percentage, REM latency, REM density, stage 1, 2, time and percentage, slow wave sleep (DELTA) time and percentage of drug-free patients with bipolar disorder were compared with case-control data of healthy controls and drug-free patients with unipolar depression. The primary outcome was the standard mean difference. Data were fitted with a random effects model. Publication bias assessment was checked by Egger’s regression and funnel plot asymmetry **Results**: Total sleep time and sleep efficiency were reduced in both manic and depressive drug-free bipolar patients compared with healthy controls. Delta sleep time and percentage were reduced only in the depressive patients, whereas the manic patients showed decreased stage 2 sleep time, reduced REM sleep time, shortened REM latency and increased REM density. Drug-free patients with unipolar depression showed reduced total sleep time and increased REM density compared with drug-free patients with bipolar depression. Drug-treated bipolar patients showed no differences compared with healthy controls, except for reduced % REM, increased REM latency and increased REM density. **Conclusions**: The results confirm the presence of sleep alterations in bipolar disorder. Although sleep duration is reduced in both manic and depressive patients, reduced delta sleep in depressive patients and increased activity/pressure of REM in manic patients appear to characterize the two phases of the illness. Altered monoaminergic activity during the depressive phase and increased cholinergic activity during the manic phase might possibly be linked to the sleep alterations also contributing to the mood changes and switch mechanisms.

## 1. Introduction

Bipolar disorder (BD) is a chronic psychiatric condition [1,2] that is estimated to affect 0.5–3% of the population [2,3,4]. Periods of depressed mood, with the hallmark loss of interest and pleasure, alternate with periods of hypomania (type II BD) or mania (type I BD), presenting with symptoms that range from unusually elated mood, disinhibition and restlessness to extreme irritation and anger [1,2,3,4,5]. Depressive episodes are nonetheless reported to account for 75% of the symptomatic time [1]. Both manic and depressive phases can present psychotic features [6], especially in those diagnosed with type I BD [7]. Psychotic symptoms can be congruent or incongruent with the mood phase, with incongruence being associated with a poorer outcome of the condition [6]. These abnormal mood periods are separated by “euthymic” intervals where mood appears normal, although residual symptoms are present in most cases [8,9]. Periods of euthymia can last weeks to years [10]. Such periods can be virtually absent in rapid-cycling patients, defined as experiencing at least four mood episodes in a year [11,12,13] and associated with poorer treatment response [14]. Similarly, BD patients with mixed features are associated with poorer treatment response and outcomes [15], experiencing overlapping symptoms of both manic/hypomanic and depressive phase [16,17,18]. Euthymia is often achieved with pharmacological treatment: despite lithium salts being the gold standard choice [19], other mood stabilizers like valproic acid [20] and lamotrigine [21] and antipsychotics like quetiapine [22,23] are widely used for the treatment of BD as well [24]. Antidepressants are generally avoided as monotherapy given their risk to trigger mania [24,25]. Nonetheless, poor compliance with pharmacological treatment is common in BD [1]. Patients with BD have the highest suicide rate among psychiatric disorders [26], with approximately 15–20% of patients dying by suicide [1,27].

Sleep disturbances in BD have been reported since early studies on the “manic-depressive illness” [28,29,30] and are associated with poorer quality of life [31,32,33] and cognitive functioning [34,35], suicide attempts [36,37] and higher mood episode relapse [38,39]. The circadian rhythm disturbances observed in BD are believed to play a pivotal role in the genesis and outcome of BD [40,41,42,43,44]. Sleep reduction has been proposed to be the common pathway of various biological and psychosocial factors triggering the onset of mania, creating a self-reinforcing cycle [45]. A decreased need for sleep and insomnia is, in fact, observed in hypomania and mania [46], whereas the depressive phase is associated with both insomnia and hypersomnia [38,47,48], although the latter appears to be more related to anergia rather than to increased sleep propensity [49]. A high prevalence of sleep disturbances has nonetheless been observed prior to a mood episode [50].

### 1.1. Polysomnographic Studies of Bipolar Disorder and Healthy Controls

Polysomnography (PSG) case-control investigations of sleep in BD and in healthy controls (HC) have been conducted in the last decades with heterogeneous results. Total sleep time (TST) was consistently found to be decreased in drug-free manic patients [51,52,53] and normal in drug-free depressed BD patients [54,55,56,57,58,59]. Nonetheless, a decreased TST in bipolar depression was also found [51,60,61]. Sleep onset latency (SOL) was found to be prolonged in bipolar depression [51,54,60,61], although other studies reported normal SOL in both the depressive [55,57,58,59] and manic/hypomanic phase [52,53,62,63]. Sleep efficiency (SE) was reported to be decreased in mania [51,53], hypomania [63] and bipolar depression [51,54,61], although several studies reported normal SE in mania [52,62] and bipolar depression [55,57,58,60]. Wake after sleep onset (WASO) was mostly reported to be normal in both mania [51,52,53,62] and bipolar depression [51,54,55,57,58,59,60].

Reports on NREM sleep in bipolar disorder are not consistent. Some studies reported no abnormalities in drug-free BD [52,62,64], while others reported abnormalities in either or both sleep stage 1 and 2 duration (ST1, ST2) and percentage (%ST1, %ST2) [53,56,57,59,60,61,63]. Regarding slow-wave sleep (SWS), several studies found no differences in either delta sleep time (DELTA) or percentage (%DELTA) regardless of the mood phase [51,52,54,58,59,61], while few studies reported reduced stage 3 sleep duration (ST3) [62] or increased percentage (%ST3) [53]. %DELTA was found to be reduced in hypomania [63].

Reports on REM sleep in bipolar disorder are similarly heterogeneous: REM sleep time (REMT) and percentage (%REM) were found to be normal regardless of the mood phase [51,53,54,56,57,58,59,60,62,63,65]. REM latency (REML) was mainly reported to be shortened in the depressive phase [51,54,55,58,60,61,66] and in mania [51,52,53] or hypomania [63], although not different in other studies [57,59,62,65,67]. REM density (REMD) was reported to be increased [52,53,58] in mania, hypomania [63] and in bipolar depression [60,61], although other studies reported normal REMD in each phase [51,54,55,57,59,65].

PSG studies in drug-free euthymic patients are scarce, reporting increased %REM and REMD in the first sleep cycle [68], increased ST1 [69] or no differences compared with HC [70]. One study reported reduced TST and increased SOL in euthymic BD [71].

### 1.2. Polysomnographic Studies of Bipolar and Unipolar Depression

Since early studies, patients with bipolar depression have been reported to have longer TST [54,55,58,60,72,73,74] and increased SE [54,55,58,73] than patients with unipolar depression (UD). Reports on SOL and WASO [51,54,55,60,73] in BD depression compared with UD, conversely, provided very heterogeneous results.

A similar heterogeneity of findings concerns both NREM sleep [51,60,73,74,75], including SWS [51,54,56,58,72,73,76], and REM sleep parameters [51,54,55,56,58,59,60,61,64,67,72,73,74,77].

A recent study reported reduced %ST2, %DELTA and REML and increased %REM in minimally treated first-episode BD compared with UD [78].

### 1.3. Polysomnographic Studies of Pharmacological Treatment in Bipolar Disorder

The effect of lithium on BD sleep parameters has been investigated in a limited number of studies, most of which reported an increase in TST and SE, a reduction in SOL but no consistent beneficial effect on WASO. NREM sleep increased, especially through an increase in ST2 and SWS, as REMT increased and REML was prolonged [79,80,81]. Some studies investigated the effect of tricyclic antidepressant amitriptyline or monoamine oxidase inhibitor tranylcypromine on bipolar depression sleep, both showing a marked REM-suppressing effect [82,83]. Regarding the effect of antipsychotic treatment on sleep during the manic phase, olanzapine was found to be more effective than haloperidol in stabilizing sleep, increasing SE and decreasing WASO [84], and quetiapine was reported to increase TST and REML [85].

When BD patients receiving stable lithium treatment were compared with HC, some studies found no effect of lithium on sleep parameters [86] or that TST, SE and NREM sleep were still decreased in BD [87]. Nonetheless, another study found increased NREM and significant REM suppression after long-term lithium treatment [88]. PSG studies of BD patients on stable combination pharmacotherapy (mood stabilizers, antipsychotics, antidepressants, benzodiazepines) reported an overall stabilization of sleep parameters, although with an increase in REMD and a longer SOL, compared with HC [89,90,91,92,93,94].

Very few reviews and metanalyses have specifically addressed sleep alterations in bipolar disorder. This appears as an important “caveat” in the understanding of the disorder considering its neurobiological specificity [95,96,97] and the connections between sleep and the illness [98,99].

In the present work, a systematic and exhaustive search strategy allowed the retrieval and consolidation of all available polysomnographic studies in bipolar disorder, including older and less accessible reports, and their re-organization into a harmonized dataset with consistent inclusion criteria. This approach enabled a methodologically homogeneous synthesis of polysomnographic evidence and supported subgroup meta-analyses by mood phase and treatment status, including drug-free manic patients, that were not assessed in prior quantitative reviews.

The sleep parameters in different phases of bipolar disorder, when drug-free or under drug treatment and in comparison with case control of healthy subjects and unipolar depressive, were reviewed and subjected to meta-analyses.

## 2. Methods

**Figure 1:**
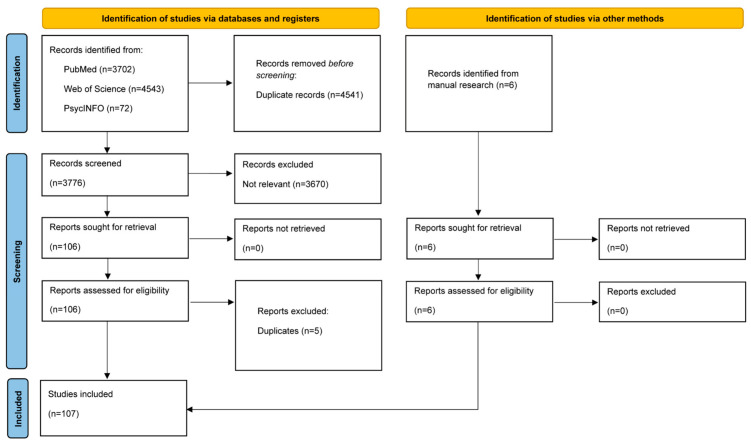
PRISMA diagram.

The literature search was conducted in PubMed, Web of Science (WoS) and PsycINFO from inception to 2026. The main search strategy combined the terms “(sleep OR sleep*) AND bipol*”. This was complemented by additional keyword blocks to ensure comprehensive coverage, including “mania OR manic OR manic-depressive”, “PSG OR polysomnogra*”, “EEG OR electroencephalogra*”, “REM OR REMS OR rapid-eye-movement OR rapid-eye-movements”, “NREM OR non-REM”, “SWS OR slow-wave”, “paradox OR paradox*”, “D-phase OR dream OR dream*”, “night OR night*”and “architect*”. To capture pharmacological effects in BD, additional terms were included in the search, such as: “mood stabilizer”, “anticonvulsant”, “lithium”, “antipsychotic”, “Depakote” (Abbott Park, IL, USA), “Lamictal” (Brentford, UK). The search yielded 3702 studies from PubMed, 4543 studies from WoS and 72 studies from PsycINFO. Out of these 8317 studies, 4541 resulted in duplicates and were removed. The remaining 3776 studies were entirely read to assess their eligibility: 3670 studies were excluded due to the absence of sleep-related data. Full-text assessment identified 106 eligible reports; 5 were excluded due to overlapping patient samples already reported in prior publications. An additional 6 eligible reports were identified through manual searching, resulting in 107 included studies. Of these, 43 were included in the meta-analyses and 64 in the systematic review only. The eligibility decision was made after a full-text article reading by the authors (GB, BT and DM), all agreeing on the inclusion or exclusion of each study. Meta-analyses were conducted for sleep parameter differences across drug-free BD vs. HC (Table 1), drug-free BD vs. drug-free UD (Table 2), treated BD vs. HC (Table 3), and pre-/post-treatment comparisons in BD (Table 4). Case-control studies included in the systematic review only are reported in Table 5 (with the relative reason for exclusion) [78,93,100,101,102,103,104,105,106,107,108,109,110,111]. All the other non-case-control studies included in the systematic review only are reported in Table 6 [29,30,49,85,112,113,114,115,116,117,118,119,120,121,122,123,124,125,126,127,128,129,130,131,132,133,134,135,136,137,138,139,140,141,142,143,144,145,146,147,148,149,150,151,152,153,154,155,156,157,158]. Two reports [85,93] contributed separate datasets, one included in meta-analysis and one in the systematic review. The present work followed the PRISMA (Preferred Reporting Items for Systematic Reviews and Meta-Analyses) guidelines, and the corresponding PRISMA flow diagram is presented in Figure 1. The PRISMA checklist is provided as Supplementary Materials (Supplementary File S1).

### 2.1. Eligibility Criteria

Studies were considered eligible for inclusion in the meta-analysis if they met the following criteria:

(1) Case-control studies including at least one sample of patients with BD, whether drug-naive, drug-free, or treated, and one HC sample.
(2) Studies including at least one sample of patients with BD and one sample of patients with UD, both of whom were drug-naive or drug-free.
(3) Studies including at least one sample of patients with BD assessed before (while drug-naive or drug-free) and after treatment with at least one antipsychotic, antidepressant or mood stabilizer.
(4) Studies in which the drug-free period was clearly reported.
(5) Studies in which BD was diagnosed according to DSM and/or ICD criteria.
(6) Studies in which sleep was assessed using PSG and scored according to the AASM or Rechtschaffen & Kales criteria.

Studies were excluded based on the following criteria:

(1) Studies without an HC sample.
(2) Studies in which the BD sample included more than 5% of participants with diagnoses other than BD (e.g., a mixed BD and UD sample).
(3) Studies in which more than 5% of the BD sample differed from the majority of the sample with respect to treatment status (e.g., 90% drug-free and 10% treated).
(4) Studies with a BD patient sample with comorbidities (e.g., ADHD, GAD).
(5) Studies in which the mood phase of the BD sample was not specified.
(6) Single-case studies.
(7) Studies describing PSG measurements in BD patients without reporting clear data.

The excluded studies were included in the systematic review.

As specified above, studies were excluded if the BD sample was not sufficiently homogeneous with respect to diagnosis and medication status. A conservative threshold (>5% of the sample) was applied to minimize potential confounding [159,160] resulting from the inclusion of participants with different diagnoses and/or exposure to psychotropic medications, both of which may substantially influence sleep architecture and polysomnographic parameters.

The quality of each study included in the systematic review and/or meta-analysis was assessed using the Newcastle–Ottawa Scale, and only studies with a score of at least 6 were included in the meta-analysis.

### 2.2. Data Extraction

Data extracted from each study included sample size, age, sex, diagnosis, medication status, medication type (when reported), and mean and standard deviation (SD) values for TST, SOL, SE, WASO, REMT, %REM, REML, REMD, ST1, ST2, ST3, ST4, %ST1, %ST2, %ST3, %ST4, DELTA and %DELTA.

In several studies, SDs for sleep variables were not reported. In these cases, SDs were estimated using a weighted average approach within each meta-analysis. SD imputation was required for approximately 20% of study-level comparisons, primarily involving ST1, ST2 and DELTA analyses. Specifically, for each outcome (e.g., REM density in BD vs. HC), a pooled SD was computed by weighting available SDs by sample size across studies. This estimated SD was then assigned to studies within the same meta-analysis that reported the corresponding mean values but omitted SDs. This approach has been previously described as acceptable for meta-analytic synthesis [161] and constituted the only imputation procedure applied in the present work. Sensitivity analyses were conducted to assess the robustness of the meta-analytic findings to SD imputation by excluding studies requiring SD imputation where feasible, and by repeating the meta-analyses after replacing imputed SDs with the minimum and maximum observed SDs from comparable studies. In a limited number of cases, missing sleep parameters were derived from other reported variables (e.g., REMT calculated as TST × %REM/100). All such derivations are explicitly documented in Supplementary Materials (Supplementary File S2).

Several studies contributed more than one dataset, either due to multiple independent patient samples or distinct diagnostic subgroups reported separately within the same publication. These were treated as independent entries in the analyses, and repeated citations reflected separate datasets rather than duplicate inclusions of the same sample.

### 2.3. Statistical Analysis

Jamovi 2.6.44 (Windows x64 desktop version) was used for all meta-analyses. Statistical power calculations were performed in R 4.6.0 (Windows x64) using the metapower 0.2.2 package. A minimum of three datasets was required for each meta-analysis. Effect sizes were computed as standardized mean differences. All analyses were conducted using a random-effects model. Between-study heterogeneity was estimated using the restricted maximum likelihood (REML) estimator, with τ^2^ reported alongside Cochran’s Q-test and the I^2^ statistic. When τ^2^ was greater than zero, prediction intervals for true effects were also calculated. Influence diagnostics included studentized residuals and Cook’s distances. Studies with studentized residuals exceeding the Bonferroni-corrected threshold based on a two-sided α = 0.05 were considered potential outliers. Studies with Cook’s distances larger than the median plus six times the interquartile range were considered influential. Publication bias was assessed using both rank correlation and regression tests, with the standard error of the observed effects used as the predictor in the latter. For meta-analyses including fewer than 10 studies, the Knapp–Hartung correction was applied to adjust standard errors and reduce the risk of inflated type I error due to small sample size.

## 3. Results

**Figure 2:**
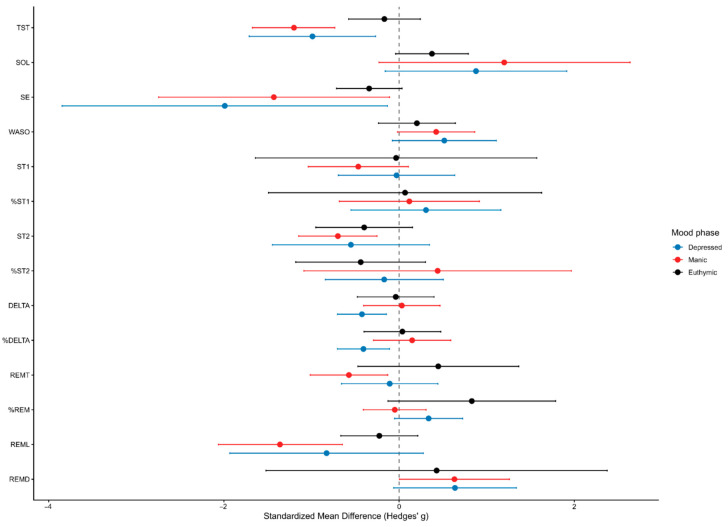
Forest plot of sleep parameter differences between drug-free BD and HC.

A total of 43 case-control studies with 670 bipolar patients, 520 healthy controls, and 280 patients with unipolar depression satisfied the criteria to be eligible for the meta-analysis [51,52,53,54,55,56,57,58,59,60,61,62,63,64,65,66,67,68,69,70,71,72,73,74,75,76,77,79,80,81,82,83,84,85,86,87,88,89,90,91,92,93,94]. The results of the extended meta-analysis are reported in Table 7. Parameters relating to heterogeneity, publication bias, the presence of outliers or particularly influential studies, and tests for possible funnel plot asymmetry are reported in Table 8. Figure 2 shows the forest plot of sleep parameter differences between drug-free BD and HC.

**Figure 3:**
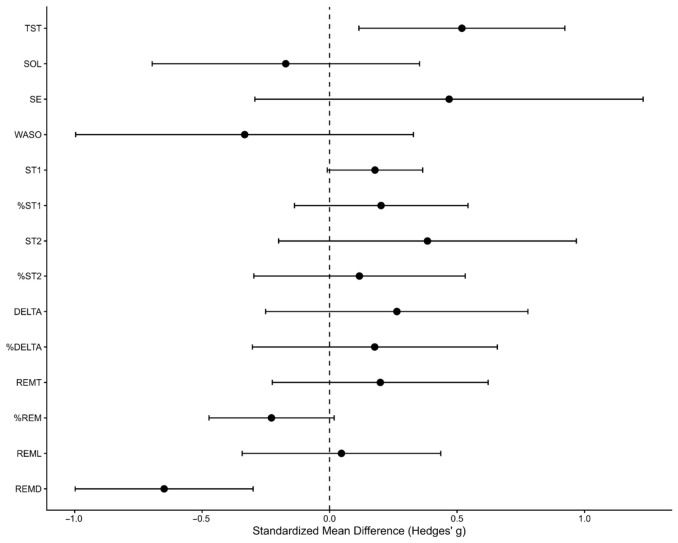
Forest plot of sleep parameter differences between drug-free depressed BD and UD.

Figure 3 shows the forest plot of sleep parameter differences between drug-free depressed BD and UD.

**Figure 4:**
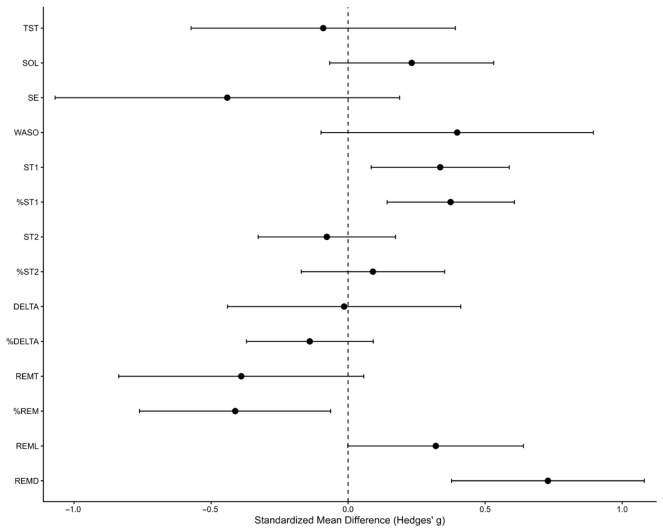
Forest plot of sleep parameter differences between treated BD and HC.

Figure 4 shows the forest plot of sleep parameter differences between treated BD and HC.

**Figure 5:**
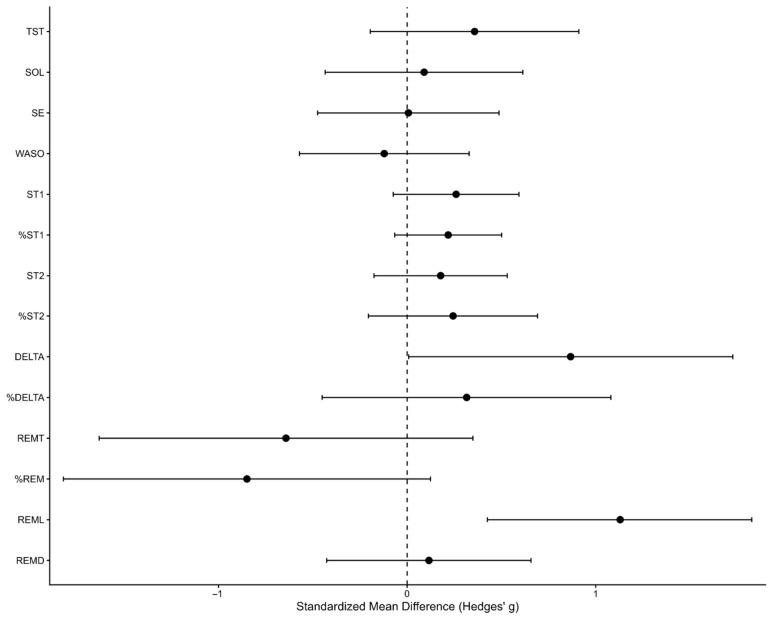
Forest plot of sleep parameter differences between pre- and post-treatment BD.

Figure 5 shows the forest plot of sleep parameter differences between pre- and post-treatment BD.

### 3.1. Sleep Duration and Continuity

The TST in drug-free BD patients, either in the depressive (369.24 ± 54.57 vs. 403.39 ± 31.39; *p* = 0.007) or manic (311.88 ± 84.76 vs. 391.24 ± 42.93; *p* = 0.001) patients, were reduced compared with HCs. Unipolar drug-free depressive patients showed a reduced TST compared with drug-free BD depressive patients (372.28 ± 61.40 vs. 348.66 ± 63.33; *p* = 0.012). Pharmacological treatment appeared to normalize the TST in patients with BD when compared with HCs (386.97 ± 73.72 vs. 391.01 ± 66.82; *p* = 0.712). The SOL of drug-free BD depressive patients did not significantly differ compared with HCs (32.53 ± 19.95 vs. 19.49 ± 10.06; *p* = 0.097) or drug-free UD patients (37.11 ± 22.09 vs. 36.23 ± 13.71; 0.520). The SE of drug-free BD patients was reduced in depressive (81.22 ± 8.18 vs. 88.60 ± 5.32; *p* = 0.036) and manic (74.76 ± 14.66 vs. 87.90 ± 5.20; *p* = 0.034) patients compared with HCs, while it did not appear to differ from that of drug-free UDs (*p* = 0.227). Pharmacological treatment appeared to normalize the SE of patients with BD (*p* = 0.169). The WASO parameter was not different in drug-free BD patients compared with HC or UD patients.

### 3.2. NREM Sleep

ST2 was reduced in drug-free BD manic patients compared with HCs (176.11 ± 95.00 vs. 229.25 ± 41.00; *p* = 0.002); DELTA and % DELTA were reduced in drug-free BD depressive patients compared with HCs, respectively (44.10 ± 34.00 vs. 68.87 ± 30.33; *p* = 0.003) and (11.98 ± 7.74 vs. 16.86 ± 6.16; *p* = 0.007). Drug treatment appeared to normalize the DELTA of treated patients with BD (59.75 ± 35.64 vs. 63.91 ± 32.15; *p* = 0.946); comparison of pre- and post-treatment DELTA values in patients with BD showed higher post-treatment values (37.01 ± 16.83 vs. 48.84 ± 25.88; *p* = 0.048). Treated BD patients had also increased ST1 (47.80 ± 30.73 vs. 39.95 ± 21.53; *p* = 0.009) and %ST1 (12.16 ± 8.54 vs. 9.63 ± 5.21; *p* = 0.002) compared with HCs.

### 3.3. REM Sleep

REMT was reduced in drug-free BD manic patients (61.48 ± 29.62 vs. 75.79 ± 18.29; *p* = 0.011) compared with HCs. Drug-treated BD patients showed a reduced %REM compared with HCs (16.47 ± 6.66 vs. 19.79 ± 7.04; *p* = 0.020). REML in drug-free BD patients, compared with HCs was reduced in manic patients (54.58 ± 15.35 vs. 75.76 ± 20.75; *p* < 0.001). Treated BD patients showed reduced %REM (16.47 ± 6.66 vs. 19.79 ± 7.04; *p* = 0.020), increased REM latency (108.43 ± 53.54 vs. 90.27 ± 40.95; *p* = 0.050) and increased REM density (12.06 ± 7.91 vs. 7.37 ± 4.92; *p* = 0.001) compared with healthy controls. Post-treatment values showed increased REML in patients with BD compared with pre-treatment values (61.67 ± 31.46 vs. 126.89 ± 61.83; *p* = 0.002).

REMD was increased in drug-free BP manic patients compared with HCs (8.14 ± 5.07 vs. 7.24 ± 0.44; *p* = 0.048). Drug-free BD depressive patients had reduced REMD compared with drug-free UD patients (1.84 ± 0.50 vs. 2.14 ± 0.49; *p* < 0.001).

## 4. Discussion

Studies on polysomnographic EEG in bipolar patients have been generally inconclusive due to small sample size, with most of the studies referring to single case reports, clinical heterogeneity of patients, different phases of the illness and medication status. Reviews and meta-analyses on sleep in bipolar disorder have also frequently combined heterogeneous assessment methods, including subjective reports, actigraphy, and polysomnography, and are limited by incomplete identification of the whole available polysomnographic literature.

A review [162] on EEG sleep alterations of bipolar patients across different clinical stages and of high-risk individuals, including a total of 22 studies, described as the most consistent findings across all stages of the disorder, increased sleep onset latency and increased REM density. Of the total sample included, 6 studies had patients during drug treatment and 14 studies during a medication-free period.

A recent meta-analysis [163] on the sleep of bipolar patients across mood phases has reported that during a depressive phase, patients had a higher percentage of REM sleep, while in a manic/mixed phase, patients exhibited shorter total sleep time, lower sleep efficiency, and longer sleep onset latency. The study did not differentiate patients according to their drug status and included sleep parameters assessed by either actigraphy, polysomnography or sleep diary. For several EEG sleep architecture variables, meta-analysis was not applicable, while for others the number of studies considered was between two and three.

The present study focuses specifically on polysomnographic evidence and includes a larger number of studies than previous meta-analyses. This allowed a more homogeneous methodological framework and phase-specific and treatment-specific meta-analyses that were not feasible in previous quantitative reviews. Findings suggest a phase-dependent pattern of sleep alterations in bipolar disorder. Slow-wave sleep reductions appear more evident in depressive episodes, whereas REM-related changes, including reduced REM latency and increased REM density, are more frequently observed in manic states. These alterations tend to be attenuated in treated and euthymic patients, consistent with a predominantly state-dependent expression rather than a stable trait.

A reduction in SWS is a consistent feature in major depression [164]. Borbely and Wirz-Justice [165] have proposed that process S is deficient in depression and that a rebound of SWS following sleep deprivation might restore SWS and improve depression.

Drug-free manic patients showed a reduction in total sleep time and sleep efficiency but delta sleep was preserved, consistent with an early EEG sleep study in unmedicated manic patients [52]. A reduced need for sleep in manic patients was confirmed by both reduced stage 2 time and reduced REM time; they also had shortened REM latency and increased REM density. An early and seminal work on rapid cycling bipolar patients [115] described that the shifts to an elevated mood were accompanied by increased REM activity (number of REMs in the REM period) during the second half of the night while the shifts to depressed mood were associated with decreased REM activity during the second half of the night; furthermore, the REM index (ratio of REM activity to REM time) was decreased on “depressive” nights. These REM alterations might indicate an increased REM pressure, possibly reflecting enhanced cholinergic activity [166,167]. A testable working hypothesis is that, in the manic phase, cholinergic mechanisms could partly contribute or correlate to mood changes and “switch” mechanisms.

Treated bipolar patients showed no differences compared with healthy controls, except for reduced % REM, increased REM latency and REM density. Although most of the treated patients were on mood stabilizers, the use of antidepressants was not negligible in the sample analyzed; thus, a reduced REM % and increased REM latency can be attributed to the well-known REM suppressing effect of antidepressants. Results of the comparison between pre- and post-treatment also showed increased REM latency following treatment.

REM density has been considered as a possible trait marker in mood disorders, also present in high-risk individuals. It has also been suggested that REM density may reflect sleep satiety [168]; Aserinsky found that, in extended sleep, REM density increased with each successive REM episode, approaching a maximum value, with no further changes with additional sleep. According to Aserinsky, REM density reflects the satisfaction of a sleep need or the build-up of a pressure to awaken. Feinberg et al. [169] first proposed that REM density may also be related to level of arousal, with REM density being lower when sleep is deeper. A recent paper has proposed REM density as a physiological marker that indicates during sleep the “time to wake” [170].

Patients with unipolar depression showed reduced total sleep time and increased REM density compared with patients with bipolar depression, Differences in the sleep of bipolar and unipolar depression suggest that EEG sleep might serve as a possible tool to differentiate the two diagnostic categories. At a clinical level, unipolar patients show more frequent insomnia, whereas bipolar patients show either insomnia or (not restorative) hypersomnia. A recent meta-analysis [171] has reported that depression in unipolar disorder was associated with a shorter total sleep time and poorer sleep efficiency than patients diagnosed with bipolar depression. From a clinical perspective, the present findings suggest that polysomnographic alterations in bipolar disorder are more closely related to current mood state than to a stable trait of the disorder, given the partial normalization of most sleep parameters in treated patients. Regarding differential diagnosis, the present meta-analysis also indicates group-level differences in REM-related parameters, particularly REM density, which was more frequently increased in unipolar depression compared with bipolar depression, although with substantial overlap and heterogeneity across studies. Nevertheless, the persistence of some alterations, particularly REM-related measures such as increased REM density in a subset of treated patients, may reflect residual physiological differences that are not fully captured by clinical remission. Further longitudinal studies are needed to clarify whether these findings have any prognostic or clinical relevance.

Although the present study addressed all the available data in the literature, some aspects should be considered when interpreting the results. The meta-analyses concerning manic and euthymic bipolar disorder samples were based on a small number of datasets, reflecting the limited availability of eligible studies and resulting in imprecise and unstable effect estimates. Consequently, statistical power was limited, with wide confidence intervals and reduced reliability of the pooled results. Several analyses also exhibited substantial heterogeneity, which constrains the robustness of quantitative inferences, although this is a frequent issue in all psychiatric samples. The imputation of missing standard deviations represents a potential source of uncertainty, as estimated SDs may affect the precision of effect size calculations and the assessment of between-study variability. However, sensitivity analyses evaluating the impact of SD imputation did not materially alter the pooled estimates, suggesting that the main findings were not substantially influenced by the imputation procedure. Nevertheless, exclusion-based sensitivity analyses for ST1, ST2 and DELTA outcomes could not be performed because too few studies with available SDs remained after excluding studies requiring imputation, preventing a reliable re-analysis. It should also be considered that a major limitation of studies assessing a biological variable in a clinical population is the medication status; drug treatment can clearly influence the measures addressed. Drug treatments in bipolar patients include mood stabilizers such as lithium and valproate, antidepressants, antipsychotics and benzodiazepine. All these classes of drugs have been shown to affect sleep. In the data analyzed, most of the drug-free patients had a wash-out period of at least two weeks, with very few studies with a shorter wash-out period. Although two weeks is the gold standard when studying a drug-free patient, a residual effect of the drugs cannot be excluded, especially when considering rebound effects on the sleep variables from the withdrawal of previous treatment (i.e., REM rebound following REM suppressing effects from antidepressants).

Finally, another limitation concerns the interpretation of PSG findings in pharmacologically treated manic patients. Sleep changes observed after treatment likely reflect both the resolution of manic symptomatology and the effects of medications on sleep architecture such as those of lithium and atypical antipsychotics. However, the included studies are few, involve very small samples, and provide limited and inconsistent information on medication class, dosage and treatment duration, preventing separation of illness-related effects from pharmacological effects. In addition, post-treatment clinical status is insufficiently characterized across studies, with no standardized reporting of residual symptoms or remission criteria, making it unfeasible to stratify PSG outcomes according to the degree of clinical recovery. As a result, a direct pre-post comparison of sleep architecture in fully remitted versus partially responsive manic patients could not be performed, and observed changes should be interpreted as reflecting a combined effect of treatment response and medication exposure rather than a pure marker of manic state normalization.

## 5. Conclusions

Several lines of evidence suggest a close relationship between mechanisms governing sleep and mood in bipolar disorder. Sleep disturbances are core clinical features either in the depressive and manic phases; sleep deprivation induces a rapid although temporary antidepressant effect in the depressive phase and can induce mania when the patient is euthymic; most of drugs used to treat the illness also have effects on sleep parameters.

The present meta-analysis confirms the presence of polysomnographic EEG sleep alterations in bipolar disorder. Although total sleep duration was reduced in both depressive and manic episodes, the two mood states showed different patterns in the main sleep components. Drug-free bipolar depression was characterized by a consistent reduction in delta sleep, whereas drug-free mania showed shortened REM latency and increased REM density. In treated patients, most sleep parameters did not differ substantially from healthy controls, suggesting that a considerable proportion of the observed abnormalities may be state dependent.

Alterations in monoaminergic and cholinergic systems have been linked to sleep architecture abnormalities in mood disorder. It has been suggested that REM sleep is promoted by cholinergic activation within the medial pontine reticular formation, while it is inhibited by the activation of serotoninergic and noradrenergic nuclei [172]. Reductions in REM latency and increased REM density could be partly explained by either impaired noradrenergic neurons in the locus coeruleus [173] or increased activity of the cholinergic system [167].

Further studies are needed to clarify the involvement of these neurotransmitter systems in the relationship between mood states, treatment effects and sleep-related abnormalities, with a specific focus also on the persistence of increased REM density even in treated patients.

## Figures and Tables

**Table 1:** Drug-free BD vs. HC studies included in the meta-analysis.

Study	N. (BD/HC)	Age (BD/HC)	Males % (BD/HC)	Diagnosis (BD/HC)	Medication Status
Duncan et al. (1979) [54]	22/36	?/?	32/44	Bipolar disorder (depressed)	Drug-free (≥2 weeks)
Feinberg et al. (1982) [55]	15/41	38.91/44	27/45	Bipolar disorder (depressed)	Drug-free (≥2 weeks)
Sitaram et al. (1982) [68]	14/15	30/26.8	43/53	Bipolar disorder (euthymia)	8 drug-free (≥2 weeks) 2 drug-free (≥12 weeks) 4 drug-naive
Linkowski et al. (1985) [64]	10/3	45/47	100/100	Bipolar disorder (depressed)	Drug-free (≥15 days)
Mendlewicz et al. (1985) [56]	8/8	44/48	100/100	Bipolar disorder (depressed)	Drug-free (≥15 days)
Linkowski et al. (1986) [51]	6/3	51/44	100/100	Bipolar disorder I (manic)	Drug-free (≥8 days)
Linkowski et al. (1986) [51]	6/3	49/44	100/100	Bipolar disorder I (depressed)	Drug-free (≥2 weeks)
Jernajczyk (1986) [65]	10/10	(26–56)/(25–54)	50/50	Bipolar disorder (depressed)	Drug-free (≥2 weeks)
Knowles et al. (1986) [70]	10/10	35.5/?	70/?	Bipolar disorder I (euthymia)	Drug-free (≥3 weeks)
Avery et al. (1986) [67]	2/11	?/52	?/36	Bipolar disorder (depressed)	Drug-free (≥6 days)
De Maertelaer et al. (1987) [60]	11/9	41/35	100/100	Bipolar disorder (depressed)	Drug-free (≥2 weeks)
Sack et al. (1988) [71]	6/6	?/?	0/0	Bipolar disorder (euthymia)	Drug-free (≥3 weeks)
Linkowski et al. (1987) [66]	5/3	47.6/47	100/100	Bipolar disorder (depressed)	Drug-free (≥2 weeks)
Hudson et al. (1988) [52]	9/18	30.4/30.1	44/44	Bipolar disorder I (manic)	Drug-free (≥8 days)
Thase et al. (1989) [57]	26/26	37.2/?	35/?	7 bipolar disorder I (depressed) 19 bipolar disorder II (depressed)	Drug-free (≥2 weeks)
Nurnberger et al. (1989) [69]	18/14	32/32	28/71	Bipolar disorder I (euthymic) Bipolar disorder II (euthymic)	Drug-free (≥1 week)
Lauer et al. (1992) [58]	10/12	36.7/36.4	?/42	Bipolar disorder I (depressed)	Drug-free (≥1 week)
Hudson et al. (1992) [53]	19/12	26.8/24.5	63/37	Bipolar disorder I (manic)	Drug-free (≥2 weeks)
Hudson et al. (1993) [61]	10/7	27.5/24.5	?/37	Bipolar disorder (depressed)	Drug-free (≥2 weeks)
Linkowski et al. (1994) [62]	8/14	41/39	100/100	Bipolar disorder (manic)	Drug-free (≥8 days)
Rao et al. (2002) [59]	5/20	?/? (adolescents)	?/?	Bipolar disorder (depressed)	Drug-free (2 weeks)
Asaad et al. (2016) [63]	20/20	38.05/36.8	65/65	Bipolar disorder II (hypomanic)	Drug-free (1 week)

?: data not reported in the original study.

**Table 2:** Drug-free BD vs. drug-free UD studies included in the meta-analysis.

Study	N. (BD/UD)	Age (BD/UD)	Males % (BD/UD)	Diagnosis (BD/UD)	Medication Status
Mendels & Chernik (1972) [72]	4/7	?/?	?/?	Bipolar disorder (depressed)/UD	Drug-free (≥1 day)
Kupfer et al. (1974) [73]	5/12	?/?	?/?	Bipolar disorder (depressed)/UD	Drug-free (≥2 weeks)
Duncan et al. (1979) [54]	22/36	?/?	32/28	Bipolar disorder (depressed)/UD	Drug-free (≥2 weeks)
Feinberg et al. (1982) [55]	15/18	38.9/57.1	27/28	Bipolar disorder (depressed)/UD	Drug-free (≥2 weeks)
Linkowski et al. (1985) [64]	10/8	45/50	100/100	Bipolar disorder (depressed)/UD	Drug-free (≥15 days)
Mendlewicz et al. (1985) [56]	8/8	44/50	100/100	Bipolar disorder (depressed)/UD	Drug-free (≥15 days)
Linkowski et al. (1986) [51]	6/3	49/44	100/100	Bipolar disorder I (depressed)/UD	Drug-free (≥2 weeks)
Giles et al. (1986) [74]	10/10	32/30.8	50/50	Bipolar disorder I (depressed)/UD	Drug-free (≥2 weeks)
Giles et al. (1986) [74]	12/12	38.4/37.3	83/83	Bipolar disorder II (depressed)/UD	Drug-free (≥2 weeks)
Avery et al. (1986) [67]	2/7	?/?	?/?	Bipolar disorder (depressed)/UD	Drug-free (≥6 days)
De Maertelaer et al. (1987) [60]	11/8	41/42	100/100	Bipolar disorder (depressed)/UD	Drug-free (≥2 weeks)
Linkowski et al. (1987) [66]	5/6	47.6/51.3	100/100	Bipolar disorder (depressed)/UD	Drug-free (≥2 weeks)
Giles et al. (1987) [77]	5/5	24/42.8	40/40	Bipolar disorder (depressed)/UD	Drug-free (≥2 weeks)
Lauer et al. (1992) [58]	10/14	36.7/40.7	?/?	Bipolar disorder I (depressed)/UD	Drug-free (≥1 week)
Hudson et al. (1993) [61]	10/7	27.5/24.4	?/37	Bipolar disorder (depressed)/UD	Drug-free (≥2 weeks)
Fossion et al. (1998) [75]	14/7	42.7/42.9	36/36	Bipolar disorder I (depressed)/UD	Drug-free (2 weeks)
Fossion et al. (1998) [75]	14/7	42.8/42.9	36/36	Bipolar disorder II (depressed)/UD	Drug-free (2 weeks)
Kerkhofs et al. (1988) [76]	23/45	?/?	?/?	Bipolar disorder I (depressed)/UD	Drug-free (2 weeks)
Kerkhofs et al. (1988) [76]	40/45	?/?	?/?	Bipolar disorder II (depressed)/UD	Drug-free (2 weeks)
Rao et al. (2002) [59]	5/19	?/? (adolescents)	?/?	Bipolar disorder (depressed)/UD	Drug-free (2 weeks)

?: data not reported in the original study.

**Table 3:** Treated BD vs. HC studies included in the meta-analysis.

Study	N. (BD/HC)	Age (BD/HC)	Males % (BD/HC)	Diagnosis (BD/HC)	Treatment
Brebbia & Altshuler (1969) [86]	3/3	?/23	100/100	Bipolar disorder (remitted)	Lithium carbonate
Bert et al. (1977) [88]	10/10	39/29	?/?	Bipolar disorder (remitted)	Lithium
Ekiert & Gogol (1983) [87]	12/24	42.6/?	50/?	Bipolar disorder (remitted)	Lithium salts
Talbot et al. (2009) [89]	28/28	33.5/37.8	14/29	24 bipolar disorder I (interepisode) 4 bipolar disorder II (interepisode)	71% mood stabilizers 82% antidepressants 50% antipsychotics 21% anxiolytics 7% sleep aids
Eidelman et al. (2010) [90]	22/22	34/40	9.1/22.7	Bipolar disorder I (interepisode) Bipolar disorder II (interepisode)	“Psychotropic medications”
Kaplan et al. (2012) [91]	27/27	33.1/38.1	14.8/29.6	23 bipolar disorder I (interepisode) 4 bipolar disorder II (interepisode)	11.1% monotherapy 70.4% mood stabilizers 81.% antidepressants 48.1% antipsychotics 22.2% anxiolytics 3.7% hypnotics 3.7% drug-free
Soehner et al. (2017) [92]	16/21	?/?	?/?	Bipolar disorder I (interepisode)	75% mood stabilizers 50% antipsychotics 75% antidepressants 20% anxiolytic 10% hypnotic 5% stimulant
Leveille et al. (2025) [93]	60/60	43.4/43.4	58/59	Bipolar disorder I (depressed) Bipolar disorder II (depressed)	96% treated (mood stabilizers, antidepressants, antipsychotics, BZD, hypnotics) 2 drug-free
Del Giudice et al. (2025) [94]	16/16	42.2/40.8	63/63	Bipolar disorder I (euthymia) Bipolar disorder II (euthymia)	62.5% lithium 56.3% antipsychotics 18.8% antidepressants 12.5% benzodiazepines 18.8% anticonvulsants

?: data not reported in the original study.

**Table 4:** Drug-free BD vs. treated BD (pre- vs. post-treatment) studies included in the meta-analysis.

Study	N. (BD/BD)	Age (BD/BD)	Males % (BD/BD)	Diagnosis (BD/BD)	Treatment
Maggini et al. (1974) [79]	8	58.2	?	3 bipolar disorder I (manic) 5 bipolar disorder II (hypomanic)	Drug-free (≥5 days) → lithium carbonate
Kupfer et al. (1974) [80]	6	32.8	17	1 bipolar disorder I (manic) 2 bipolar disorder II (hypomanic) 2 bipolar disorder (depressed) 1 bipolar disorder (unclear)	Drug-free (≥2 weeks) → lithium carbonate
Ekiert & Gogol (1983) [82]	10	42.7	50	Bipolar disorder (depressed)	Drug-free (≥2 weeks) → amitriptyline
Hudson et al. (1989) [81]	5	33	25	Bipolar disorder I (manic)	Drug-free (≥8 days) → lithium
Jindal et al. (2003) [83]	23	38.6	35	6 bipolar disorder I (depressed) 17 bipolar disorder II (depressed)	Drug-free (2 weeks) → tranylcypromine
Moreno et al. (2007) [84]	5	39.2	0	Bipolar disorder I (manic)	Drug-free (≥4 days) → haloperidol
Moreno et al. (2007) [84]	7	38.6	43	Bipolar disorder I (manic)	Drug-free (≥4 days) → olanzapine
Cohrs et al. (2010) [85]	3	38	67	Bipolar disorder I (manic)	Drug-free (?) → quetiapine

?: data not reported in the original study.

**Table 5:** Case-control studies included in the systematic review only.

Study (Excluded)	N. (BD)/N. (CTRL)	Age (BD/CTRL)	Males % (BD/CTRL)	Diagnosis (BD/CTRL)	Treatment	Reason of Exclusion
Kupfer et al. (1970) [100]	7	44.9	29.00	6 bipolar disorder (?) + 1 psychotic depression	Drug-free (≥10 days) → lithium carbonate	Mixed sample
De Barros-Ferreira (1973) [101]	3/2	35.7/31	?/?	3 bipolar disorder (manic?) /HC	1° gen. antipsychotics	No mood stabilizers
Gerner et al. (1979) [102]	10/9	45/36	?/?	? Bipolar disorder (depressed) + ? UD/HC	Drug-free (≥1 week)	Unknown size, mixed sample
Ekiert & Gogol (1981) [103]	9/9	42.1/42.3	56/56	5 bipolar disorder (depressed) + 4 UD/HC	Drug-free (≥2 weeks)	Mixed sample
Mendelson et al. (1987) [104]	8/8	43.9/?	13/13	7 bipolar disorder (depressed) + 1 UD/HC	Drug-free (≥2 weeks)	Mixed sample
Armitage et al. (2004) [105]	14	40.9	29.00	11 bipolar disorder (?) + 3 schizoaffective disorder	Mood stabilizers + BZD → mood stabilizers + clozapine + BZD	Change of treatment
Bernert et al. (2017) [106]	24/30	44.4/46.1	29.7/50	Bipolar disorder (depressed)/UD	Mood stabilizers /drug-free	Mixed treatment status
Estrada-Prat et al. (2019) [107]	13/13	13.9/13.9	53.8/53.8	Bipolar disorder (euthymia) + ADHD + GAD + DBD/HC	?	Comorbidities/ unclear treatment status
Okada et al. (2022) [108]	43/81	50.6/53.3	58/62	Bipolar disorder (?)/UD	Mood stabilizers + antipsychotics + antidepressants + BZD	Both groups treated
Xu et al. (2024) [109]	19/44	20/48.3	26.3/15.8	Bipolar disorder (manic)/UD	?	Unclear treatment status
Xiu et al. (2024) [78]	107/256	22/23	40/86	83 bipolar disorder I (first episode) 24 bipolar disorder II (first episode)	Drug-free (?)	Unclear treatment status
Leveille et al. (2025) [93]	60/60	43.4/43.2	30/32	Bipolar disorder (depressed)/UD	96% treated (mood stabilizers, antidepressants, antipsychotics, BZD, hypnotics) 2 drug-free	Both groups treated
Salmeron et al. (2026) [110]	23/43	?/?	?/?	Bipolar disorder (depressed)/UD	Mood stabilizers + antipsychotics + antidepressants + hypnotics + anxiolytics	Both groups treated
Ma et al. (2026) [111]	45/10	48.8/37.4	20/40	27 bipolar disorder (depressed) + 18 UD/HC	Drug-free (2 weeks)	Mixed sample

?: data not reported in the original study.

**Table 6:** Non-case-control studies included in the systematic review only.

Study	N (Age, Phase/Diagnosis)	Treatment
Hartmann et al. (1966) [29]	1M (39, depressed)	Drug-free (1 week)
Hartmann et al. (1968) [30]	1M (39, euthymia)	Drug-free (1 week)
Hawkins et al. (1968) [112]	1M (45, hypomanic)	Drug-free (?)
Meltzer et al. (1970) [113]	1F (19, manic)	Drug-free (≥2 weeks)
Mendels & Hawkins (1971) [114]	1M (45, hypomanic)	Drug-free (1 week)
Kupfer & Heninger (1972) [115]	1M (70, depressed)	Drug-free (>10 days)
Kupfer & Heninger (1972) [115]	1M (70, manic)	Drug-free (>10 days)
Gillin et al. (1974) [116]	1F (43, depressed)	Drug-free (≥2 weeks)
Gillin et al. (1974) [116]	1F (43, hypomanic)	Drug-free (≥2 weeks)
Chernik et al. (1974) [117]	1M (37, mixed state)	Drug-free (≥8 weeks)
Chernik et al. (1974) [117]	1M (55, depressed)	Drug-free (≥8 weeks)
Schilkrut et al. (1975) [118]	1F (26, depressed)	Drug-free (≥3 weeks)
Gillin et al. (1977) [119]	1F (36, depressed)	Drug-free (15 days)
Gillin et al. (1977) [119]	1F (36, manic)	Drug-free (15 days)
Post et al. (1977) [120]	1F (39, depressed)	Drug-free (≥2 weeks)
Post et al. (1977) [120]	1F (39, manic)	Drug-free (≥2 weeks)
Jovanovic et al. (1977) [121]	22M, 38F (28.1, depressed)	Drug-free (?)
Knowles et al. (1979) [122]	1M (38, euthymia)	Drug-free (2 weeks)
Wehr et al. (1985) [123]	1F (48, depressed)	Drug-free (12 weeks)
Wehr et al. (1985) [123]	1F (48, manic)	Drug-free (12 weeks)
Wehr et al. (1985) [123]	1F (36, depressed)	Drug-free (3 weeks)
Wehr et al. (1985) [123]	1F (56, manic)	Clorgyline
Hanna et al. (1986) [124]	1M (53, depressed)	Drug-free (?)
Hanna et al. (1986) [124]	1M (53, manic)	Drug-free (?)
Campbell et al. (1989) [125]	1M (45, hypomanic)	Drug-free (>6 weeks)
Cohrs et al. (2010) [85]	1M (39, depressed)	Drug-free (?)
Zarcone et al. (1967) [126]	1M (44, depressed)	Phenothiazines/amitriptyline
Zarcone et al. (1967) [126]	1M (49, depressed)	Phenothiazines/amitriptyline
Hartmann et al. (1968) [30]	1F (48, depressed)	Chlorpromazine
Hartmann et al. (1968) [30]	1F (48, euthymia)	Chlorpromazine
Hartmann et al. (1968) [30]	1F (48, manic)	Chlorpromazine
Hartmann et al. (1968) [30]	1F (60, depressed)	Chlorpromazine
Hartmann et al. (1968) [30]	1F (60, euthymia)	Chlorpromazine
Hartmann et al. (1968) [30]	1F (60, manic)	Chlorpromazine
Hartmann et al. (1968) [30]	1F (60, depressed)	Chlorpromazine
Hartmann et al. (1968) [30]	1F (60, euthymia)	Chlorpromazine
Hartmann et al. (1968) [30]	1F (60, manic)	Chlorpromazine
De Barros-Ferreira (1969) [127]	1M (42, mixed state)	Haloperidol + chlorpromazine
De Barros-Ferreira (1969) [127]	1F (35, mixed state)	Haloperidol + chlorpromazine
Hartmann et al. (1968) [30]	1M (58, depressed)	Drug-free + ECT
Hartmann et al. (1968) [30]	1M (58, euthymia)	Drug-free + ECT
Hartmann et al. (1968) [30]	1M (58, manic)	Drug-free + ECT
Hartmann et al. (1968) [30]	1F (31, depressed)	Drug-free + ECT
Hartmann et al. (1968) [30]	1F (31, euthymia)	Drug-free + ECT
Wehr et al. (1979) [128]	1F (57, depressed)	Drug-free + ECT
Kupfer et al. (1972) [129]	3F, 1M (depressed)	Lithium carbonate
Chernik et al. (1974) [117]	1M (37, mixed state)	Lithium carbonate
Chernik et al. (1974) [117]	1M (55, depressed)	Lithium carbonate
Campbell et al. (1989) [125]	1M (45, hypomanic)	Lithium carbonate
Campbell et al. (1989) [125]	1M (45, hypomanic)	Lithium carbonate + desipramine
Nakamura et al. (1993) [130]	1F (44, manic)	Lithium carbonate + levomepromazine + alprazolam + flunitrazepam
Nakamura et al. (1993) [130]	1F (44, euthymia)	Lithium carbonate + levomepromazine + alprazolam + flunitrazepam
Fukuyama et al. (1993) [131]	1F (61, depressed)	Lithium carbonate + flunitrazepam
Fukuyama et al. (1993) [131]	1F (61, manic)	Lithium carbonate + flunitrazepam
Fukuyama et al. (1993) [131]	1F (61, euthymia)	Lithium carbonate + flunitrazepam
Riemann et al. (1993) [132]	1M (64, ?)	Carbamazepine
Meltzer et al. (1970) [113]	1M (?, ?)	Drug-free (≥2 weeks)
Sack et al. (1988) [133]	6F (?, ?)	Drug-free (3 weeks)
Nofzinger et al. (1991) [49]	10M, 15F (36.6, depressed)	Drug-free (2 weeks)
Riemann et al. (1993) [132]	1M (64, ?)	Drug-free (2 weeks)
Mehl et al. (2006) [134]	7M, 6F (6.7, pediatric BD)	?
Cohrs et al. (2010) [85]	1M (39, depressed)	Quetiapine
Pacchioni et al. (2023) [135]	17? (?, manic)	Mood stabilizers + antipsychotics + BZD
Lazowski et al. (2014) [136]	5? (?, depressed) + 5 UD	Mood stabilizers + antipsychotics + BZD
Lazowski et al. (2014) [136]	8? (?, depressed) + 7 UD	Mood stabilizers + antipsychotics + BZD
Post et al. (1974) [137]	3M (?, depressed) + 2 UD	Drug-free (≥1 week)
Chernik & Mendels (1974) [138]	8M (?, depressed) + 2 UD	Drug-free (≥2 weeks)
Chernik & Mendels (1974) [138]	8M (?, depressed) + 2 UD	Drug-free (≥2 weeks)
Post et al. (1978) [139]	3M, 4F (42.7, depressed) + 4 UD	Drug-free (≥2 weeks)
Silberman et al. (1981) [140]	28? (?, depressed) + 16 UD	Drug-free (≥2 weeks)
Kerkhofs et al. (1986) [141]	16? (?, depressed) + 11 UP	?
Sack et al. (1988) [133]	8? (?, depressed) + 6 UD	Drug-free (≥2 weeks)
Mieczkowski et al. (2014) [142]	3M, 3F (15.1, pediatric BD + OSA + ADHD + GAD)	Antidepressants + antipsychotics + mood stabilizers
Mieczkowski et al. (2014) [142]	14M, 7F (13.3, pediatric BD + ADHD + GAD)	Antidepressants + antipsychotics + mood stabilizers + stimulants
Sarzetto et al. (2025) [143]	13M, 22F (55.5, 15 BPI, 6 BPII) + 14 UD	Antidepressants + antipsychotics + mood stabilizers + hypnotics/anxiolytics
De Barros-Ferreira (1969) [127]	1M (42, mixed state)	Drug-free (?)
De Barros-Ferreira (1969) [127]	1F (35, mixed state)	Drug-free (?)
Bunney Jr. et al. (1970) [144]	3? (?, rapid cycling)	Imipramine
Shirakura (1973) [145]	1M, 2F (?, depressed)	? → Isocarboxazid
Schreiner et al. (2001) [146]	1M (81, rapid cycling)	Drug-free (?)
Berger et al. (1982) [147]	6M, 2F (26.9, depressed) + 12 UD	Drug-free (≥1 week)
Feinberg & Carroll (1984) [148]	25? (?, depressed)	Drug-free (2 weeks)
Ansseau et al. (1985) [149]	9? (?, depressed)	Drug-free (2 weeks)
Welsh et al. (1986) [150]	1F (35, rapid cycling)	Drug-free (≥5 days) → Lithium carbonate
Kumar et al. (1987) [151]	9? (?, depressed) + 17 UD	Drug-free (2 weeks)
Kumar et al. (1987) [151]	11? (?, depressed) + 19 UD	Drug-free (2 weeks)
Sholomskas et al. (1988) [152]	1F (67, hypomanic + narcolepsy)	Drug-free (?)
Gann et al. (1993) [153]	1M (64, rapid cycling)	Drug-free (?) → Carbamazepine
Fossion et al. (1994) [154]	68? (43, depressed)	Drug-free (15 days)
Lauer et al. (1995) [155]	7? (?, ?) + 11 UD	Drug-free (≥1 week)
Rush et al. (1997) [156]	15? (?, depressed) + 49 UD	Drug-free (≥2 weeks)
Voderholtzer et al. (2002) [157]	1M (40, rapid cycling)	Drug-free (?) → Lithium
De Silva et al. (2006) [158]	1M (47, rapid cycling)	Drug-free (?)

?: data not reported in the original study.

**Table 7:** Meta-analysis results.

	Meta-Analysis				
TST	N. of studies	N. of subjects	Mean ± SD	RE Model	*p*-value
Drug-free BD (depressed) vs. HC	10	125 vs. 172	369.24 ± 54.57 vs. 403.39 ± 31.39	−0.989 (−1.711 to −0.267)	**0.007**
Drug-free BD (manic) vs. HC	4	42 vs. 47	311.88 ± 84.76 vs. 391.24 ± 42.93	−1.20 (−1.675 to −0.735)	**<0.001**
Drug-free BD (euthymic) vs. HC	4	48 vs. 45	360.33 ± 71.02 vs. 378.95 ± 45.98	−0.168 (−0.577 to 0.242)	0.422
Drug-free BD (depressed) vs. UD	16	211 vs. 258	372.28 ± 61.40 vs. 348.66 ± 63.33	0.519 (0.115 to 0.923)	**0.012**
Treated BD vs. HC	7	144 vs. 161	386.97 ± 73.72 vs. 391.01 ± 66.82	−0.0908 (−0.573 to 0.391)	0.712
Pre- vs. post-treatment	8	67 vs. 67	344.10 ± 69.51 vs. 361.97 ± 70.41	−0.358 (−0.911 to 0.195)	0.204
SOL	N. of studies	N. of subjects	Mean ± SD	RE Model	*p*-value
Drug-free BD (depressed) vs. HC	8	105 vs. 154	32.53 ± 19.95 vs. 19.49 ± 10.06	0.878 (−0.158 to 1.914)	0.097
Drug-free BD (manic) vs. HC	5	62 vs. 67	37.65 ± 28.95 vs. 22.08 ± 11.45	1.20 (−0.230 to 2.636)	0.100
Drug-free BD (euthymic) vs. HC	4	48 vs. 45	32.9 ± 27.74 vs. 21.81 ± 18.62	0.375 (−0.039 to 0.790)	0.076
Drug-free BD (depressed) vs. UD	8	84 vs. 117	37.11 ± 22.09 vs. 36.23 ± 13.71	−0.172 (−0.696 to 0.3529)	0.520
Treated BD vs. HC	4	83 vs. 95	22.86 ± 28.68 vs. 15.34 ± 18.25	0.232 (−0.067 to 0.531)	0.128
Pre- vs. post-treatment	7	64 vs. 64	23.94 ± 23.77 vs. 26.39 ± 26.58	−0.0903 (−0.614 to 0.434)	0.735
SE	N. of studies	N. of subjects	Mean ± SD	RE Model	*p*-value
Drug-free BD (depressed) vs. HC	7	100 vs. 134	81.22 ± 8.18 vs. 88.60 ± 5.32	−1.99 (−3.847 to −0.132)	**0.036**
Drug-free BD (manic) vs. HC	5	62 vs. 67	74.76 ± 14.66 vs. 87.90 ± 5.20	−1.43 (−2.746 to −0.109)	**0.034**
Drug-free BD (euthymic) vs. HC	3	42 vs. 39	86.39 ± 9.63 vs. 89.78 ± 7.46	−0.342 (−0.716 to 0.033)	0.059
Drug-free BD (depressed) vs. UD	11	164 vs. 210	79.47 ± 9.92 vs. 74.04 ± 12.43	0.469 (−0.293 to 1.230)	0.227
Treated BD vs. HC	4	115 vs. 127	84.47 ± 9.76 vs. 86.85 ± 9.14	−0.441 (−1.069 to 0.188)	0.169
Pre- vs. post-treatment	6	56 vs. 56	83.21 ± 22.95 vs. 83.48 ± 13.08	−0.0072 (−0.488 to 0.474)	0.976
WASO	N. of studies	N. of subjects	Mean ± SD	RE Model	*p*-value
Drug-free BD (depressed) vs. HC	8	105 vs. 155	42.37 ± 31.40 vs. 27.42 ± 18.49	0.516 (−0.077 to 1.110)	0.088
Drug-free BD (manic) vs. HC	4	42 vs. 47	33.61 ± 37.59 vs. 30.54 ± 21.83	0.423 (−0.018 to 0.864)	0.060
Drug-free BD (euthymic) vs. HC	3	42 vs. 39	16.68 ± 24.44 vs. 12.33 ± 15.91	0.203 (−0.235 to 0.642)	0.364
Drug-free BD (depressed) vs. UD	11	116 vs. 139	58.14 ± 28.16 vs. 56.38 ± 30.11	−0.333 (−0.996 to 0.329)	0.324
Treated BD vs. HC	5	131 vs. 148	54.91 ± 43.61 vs. 44.06 ± 36.29	0.398 (−0.099 to 0.895)	0.116
Pre- vs. post-treatment	7	64 vs. 64	45.36 ± 48.23 vs. 37.52 ± 31.76	0.121 (−0.329 to 0.570)	0.598
ST1	N. of studies	N. of subjects	Mean ± SD	RE Model	*p*-value
Drug-free BD (depressed) vs. HC	5	58 vs. 65	42.92 ± 36.52 vs. 35.06 ± 18.33	−0.0291 (−0.694 to 0.636)	0.932
Drug-free BD (manic) vs. HC	4	42 vs. 47	25.06 ± 24.00 vs. 38.85 ± 28.00	−0.466 (−1.038 to 0.106)	0.111
Drug-free BD (euthymic) vs. HC	2	28 vs. 24	25.44 ± 10.6 vs. 29.96 ± 28.60	−0.0343 (−1.640 to 1.572)	0.966
Drug-free BD (depressed) vs. UD	9	87 vs. 85	56.49 ± 63.37 vs. 38.22 ± 15.9	0.178 (−0.009 to 0.365)	0.060
Treated BD vs. HC	6	117 vs. 134	47.80 ± 30.73 vs. 39.95 ± 21.53	0.336 (0.084 to 0.588)	**0.009**
Pre- vs. post-treatment	7	64 vs. 64	26.66 ± 21.83 vs. 32.49 ± 15.94	−0.260 (−0.593 to 0.073)	0.104
%ST1	N. of studies	N. of subjects	Mean ± SD	RE Model	*p*-value
Drug-free BD (depressed) vs. HC	5	58 vs. 65	11.67 ± 6.07 vs. 8.88 ± 4.17	0.307 (−0.549 to 1.162)	0.482
Drug-free BD (manic) vs. HC	5	62 vs. 67	7.41 ± 3.05 vs. 8.32 ± 3.36	0.117 (−0.682 to 0.916)	0.774
Drug-free BD (euthymic) vs. HC	2	28 vs. 24	6.89 ± 5.53 vs. 7.60 ± 4.44	0.0681 (−1.492 to 1.628)	0.923
Drug-free BD (depressed) vs. UD	9	87 vs. 85	14.49 ± 8.84 vs. 11.00 ± 5.09	0.202 (−0.138 to 0.543)	0.244
Treated BD vs. HC	7	139 vs. 156	12.16 ± 8.54 vs. 9.63 ± 5.21	0.374 (0.142 to 0.607)	**0.002**
Pre- vs. post-treatment	7	64 vs. 64	8.05 ± 4.57 vs. 8.93 ± 4.22	−0.218 (−0.502 to 0.065)	0.108
ST2	N. of studies	N. of subjects	Mean ± SD	RE Model	*p*-value
Drug-free BD (depressed) vs. HC	5	58 vs. 65	207.11 ± 46.32 vs. 228.60 ± 38.43	−0.550 (−1.447 to 0.347)	0.229
Drug-free BD (manic) vs. HC	4	42 vs. 47	176.11 ± 95.00 vs. 229.25 ± 41.00	−0.699 (−1.146 to −0.253)	**0.002**
Drug-free BD (euthymic) vs. HC	2	28 vs. 24	185.95 ± 60.70 vs. 209.09 ± 47.90	−0.3989 (−0.951 to 0.153)	0.157
Drug-free BD (depressed) vs. UD	9	87 vs. 85	183.21 ± 50.38 vs. 186.36 ± 20.16	0.384 (−0.200 to 0.968)	0.198
Treated BD vs. HC	6	117 vs. 134	207.25 ± 58.20 vs. 211.92 ± 49.64	−0.0777 (−0.328 to 0.173)	0.543
Pre- vs. post-treatment	7	64 vs. 64	202.31 ± 51.44 vs. 214.53 ± 55.31	−0.178 (−0.531 to 0.175)	0.323
%ST2	N. of studies	N. of subjects	Mean ± SD	RE Model	*p*-value
Drug-free BD (depressed) vs. HC	5	58 vs. 65	55.89 ± 7.97 vs. 55.84 ± 8.34	−0.170 (−0.843 to 0.503)	0.621
Drug-free BD (manic) vs. HC	5	62 vs. 67	55.09 ± 8.05 vs. 56.05 ± 4.73	0.440 (−1.087 to 1.968)	0.468
Drug-free BD (euthymic) vs. HC	2	28 vs. 24	55.84 ± 10.06 vs. 57.58 ± 6.03	−0.438 (−1.179 to 0.302)	0.246
Drug-free BD (depressed) vs. UD	9	87 vs. 85	47.31 ± 10.68 vs. 49.42 ± 9.89	0.117 (−0.297 to 0.532)	0.579
Treated BD vs. HC	7	139 vs. 156	55.25 ± 11.79 vs. 53.75 ± 8.64	0.0905 (−0.171 to 0.352)	0.497
Pre- vs. post-treatment	7	64 vs. 64	56.64 ± 9.62 vs. 58.93 ± 10.82	−0.244 (−0.692 to 0.205)	0.198
DELTA	N. of studies	N. of subjects	Mean ± SD	RE Model	*p*-value
Drug-free BD (depressed) vs. HC	8	100 vs. 121	44.10 ± 34.00 vs. 68.87 ± 30.33	−0.424 (−0.704 to −0.145)	**0.003**
Drug-free BD (manic) vs. HC	4	42 vs. 47	47.91 ± 34.00 vs. 45.57 ± 33.00	0.0313 (−0.405 to 0.467)	0.888
Drug-free BD (euthymic) vs. HC	3	42 vs. 39	49.64 ± 32.70 vs. 53.27 ± 22.40	−0.0384 (−0.476 to 0.399)	0.864
Drug-free BD (depressed) vs. UD	15	196 vs. 240	53.60 ± 25.33 vs. 42.36 ± 20.30	0.264 (−0.251 to 0.778)	0.315
Treated BD vs. HC	6	117 vs. 134	59.75 ± 35.64 vs. 63.91 ± 32.15	−0.0146 (−0.440 to 0.411)	0.946
Pre- vs. post-treatment	7	64 vs. 64	37.01 ± 16.83 vs. 48.84 ± 25.88	−0.867 (−1.727 to −0.008)	**0.048**
%DELTA	N. of studies	N. of subjects	Mean ± SD	RE Model	*p*-value
Drug-free BD (depressed) vs. HC	8	100 vs. 121	11.98 ± 7.74 vs. 16.86 ± 6.16	−0.407 (−0.704 to −0.110)	**0.007**
Drug-free BD (manic) vs. HC	5	62 vs. 67	16.71 ± 6.73 vs. 15.08 ± 11.06	0.149 (−0.292 to 0.590)	0.507
Drug-free BD (euthymic) vs. HC	3	42 vs. 39	14.26 ± 9.43 vs. 14.10 ± 7.08	0.0374 (−0.401 to 0.476)	0.867
Drug-free BD (depressed) vs. UD	15	196 vs. 240	14.51 ± 14.93 vs. 11.57 ± 8.42	0.177 (−0.303 to 0.658)	0.469
Treated BD vs. HC	7	139 vs. 156	15.51 ± 10.60 vs. 16.63 ± 7.99	−0.140 (−0.371 to 0.092)	0.236
Pre- vs. post-treatment	8	67 vs. 67	12.70 ± 7.74 vs. 13.84 ± 9.81	−0.316 (−1.081 to 0.450)	0.419
REMT	N. of studies	N. of subjects	Mean ± SD	RE Model	*p*-value
Drug-free BD (depressed) vs. HC	9	110 vs. 131	80.06 ± 17.38 vs. 84.50 ± 13.51	−0.107 (−0.657 to 0.443)	0.703
Drug-free BD (manic) vs. HC	4	42 vs. 47	61.48 ± 29.62 vs. 75.79 ± 18.29	−0.573 (−1.015 to −0.130)	**0.011**
Drug-free BD (euthymic) vs. HC	3	42 vs. 39	90.06 ± 26.40 vs. 80.91 ± 19.60	0.447 (−0.471 to 1.366)	0.171
Drug-free BD (depressed) vs. UD	13	133 vs. 150	79.43 ± 24.26 vs. 81.88 ± 16.17	0.199 (−0.225 to 0.622)	0.358
Treated BD vs. HC	6	117 vs. 134	62.73 ± 30.98 vs. 77.71 ± 31.56	−0.390 (−0.837 to 0.057)	0.087
Pre- vs. post-treatment	7	64 vs. 64	73.20 ± 24.74 vs. 57.14 ± 23.32	0.642 (−0.349 to 1.633)	0.204
%REM	N. of studies	N. of subjects	Mean ± SD	RE Model	*p*-value
Drug-free BD (depressed) vs. HC	9	110 vs. 131	21.38 ± 5.03 vs. 20.99 ± 3.12	0.337 (−0.051 to 0.725)	0.089
Drug-free BD (manic) vs. HC	5	62 vs. 67	20.24 ± 4.66 vs. 20.23 ± 2.80	−0.0491 (−0.407 to 0.308)	0.788
Drug-free BD (euthymic) vs. HC	3	42 vs. 39	24.92 ± 5.65 vs. 20.81 ± 4.78	0.830 (−0.128 to 1.788)	0.065
Drug-free BD (depressed) vs. UD	13	133 vs. 150	20.63 ± 5.48 vs. 22.91 ± 4.53	−0.228 (−0.473 to 0.018)	0.069
Treated BD vs. HC	6	123 vs. 135	16.47 ± 6.66 vs. 19.79 ± 7.04	−0.412 (−0.761 to −0.064)	**0.020**
Pre- vs. post-treatment	7	64 vs. 64	19.91 ± 4.79 vs. 17.58 ± 6.02	0.849 (−0.124 to 1.822)	0.087
REML	N. of studies	N. of subjects	Mean ± SD	RE Model	*p*-value
Drug-free BD (depressed) vs. HC	13	142 vs. 197	64.91 ± 44.70 vs. 81.95 ± 25.95	−0.828 (−1.933 to 0.277)	0.142
Drug-free BD (manic) vs. HC	5	62 vs. 67	54.58 ± 15.35 vs. 75.76 ± 20.75	−1.36 (−2.064 to −0.647)	**<0.001**
Drug-free BD (euthymic) vs. HC	3	42 vs. 39	74.60 ± 33.40 vs. 79.98 ± 29.21	−0.226 (−0.666 to 0.213)	0.313
Drug-free BD (depressed) vs. UD	19	229 vs. 273	61.86 ± 43.76 vs. 55.55 ± 32.73	0.0466 (−0.343 to 0.436)	0.815
Treated BD vs. HC	4	72 vs. 84	108.43 ± 53.54 vs. 90.27 ± 40.95	0.320 (−0.001 to 0.640)	**0.050**
Pre- vs. post-treatment	8	67 vs. 67	61.67 ± 31.46 vs. 126.89 ± 61.83	−1.13 (−1.828 to −0.426)	**0.002**
REMD	N. of studies	N. of subjects	Mean ± SD	RE Model	*p*-value
Drug-free BD (depressed) vs. HC	9	115 vs. 164	2.29 ± 0.86 vs. 1.85 ± 0.56	0.638 (−0.062 to 1.339)	0.074
Drug-free BD (manic) vs. HC	4	54 vs. 53	8.14 ± 5.07 vs. 7.24 ± 0.44	0.632 (0.004 to 1.259)	**0.048**
Drug-free BD (euthymic) vs. HC	2	32 vs. 29	1.7 ± 0.65 vs. 1.45 ± 0.52	0.4284 (−1.520 to 2.376)	0.218
Drug-free BD (depressed) vs. UD	10	106 vs. 139	1.84 ± 0.50 vs. 2.14 ± 0.49	−0.649 (−0.998 to −0.300)	**<0.001**
Treated BD vs. HC	3	66 vs. 66	12.06 ± 7.91 vs. 7.37 ± 4.92	0.729 (0.377 to 1.081)	**<0.001**
Pre- vs. post-treatment	5	46 vs. 46	1.17 ± 0.27 vs. 1.26 ± 0.49	−0.116 (−0.657 to 0.426)	0.675

Statistically significant *p*-value in bold.

**Table 8:** Heterogeneity, publication bias and funnel plot asymmetry analysis.

	Heterogeneity			Publication Bias				Funnel Plot Asymmetry	
TST	Q	I^2^	*p*-value	Fail-safe N	*p*-value	Outlier	Overly influential	Rank correlation (*p*-value)	Regression test (*p*-value)
Drug-free BD (depressed) vs. HC	66.772	85.79%	**<0.001**	145	**<0.001**	Duncan et al. (1979) [54]	Duncan et al. (1979) [54]	0.0466 *	0.2848
Drug-free BD (manic) vs. HC	1.317	0.00%	0.725	32	**<0.001**	-	-	0.3333	0.9804
Drug-free BD (euthymic) vs. HC	1.133	0.00%	0.769	0	0.199	-	-	1	0.76
Drug-free BD (depressed) vs. UD	55.747	73.84%	**<0.001**	123	**<0.001**	Duncan et al. (1979) [54]	Duncan et al. (1979) [54]	0.5056	0.9856
Treated BD vs. HC	20.455	71.94%	**0.002**	0	0.218	-	-	0.3813	0.7937
Pre- vs. post-treatment	15.666	53.96%	**0.028**	1	**0.041**	-	Maggini et al. (1974) [79]	0.1789	0.0070 *
SOL	Q	I^2^	*p*-value	Fail-safe N	*p*-value	Outlier	Overly influential	Rank correlation (*p*-value)	Regression test (*p*-value)
Drug-free BD (depressed) vs. HC	73.08	91.85%	**<0.001**	68	**<0.001**	Duncan et al. (1979) [54]	Duncan et al. (1979) [54]	0.2751	0.5093
Drug-free BD (manic) vs. HC	39.03	91.42%	**<0.001**	43	**<0.001**	Asaad et al. (2016) [63]	Asaad et al. (2016) [63]	0.8167	0.4907
Drug-free BD (euthymic) vs. HC	1.452	0.00%	0.484	0	0.104	-	-	1	0.6896
Drug-free BD (depressed) vs. UD	17.484	64.15%	**0.015**	0	0.151	Kupfer et al. (1974) [73]	-	0.5484	0.6367
Treated BD vs. HC	2.043	0.00%	0.563	0	**0.05**	-	-	0.3333	0.3328
Pre- vs. post-treatment	11.524	48.18%	0.073	0	0.302	-	-	0.5619	0.3322
SE	Q	I^2^	*p*-value	Fail-safe N	*p*-value	Outlier	Overly influential	Rank correlation (*p*-value)	Regression test (*p*-value)
Drug-free BD (depressed) vs. HC	104.484	96.72%	**<0.001**	148	**<0.001**	Duncan et al. (1979) [54]	Duncan et al. (1979) [54]	0.0107 *	0.0198 *
Drug-free BD (manic) vs. HC	32.325	89.54%	**<0.001**	66	**<0.001**	Asaad et al. (2016) [63]	Asaad et al. (2016) [63]	0.4833	0.5932
Drug-free BD (euthymic) vs. HC	0.3	0.00%	0.861	0	0.06	-	-	1	0.6948
Drug-free BD (depressed) vs. UD	60.229	90.72%	**<0.001**	58	**<0.001**	Hudson et al. (1993) [61]	-	1	0.714
Treated BD vs. HC	13.961	79.82%	**0.003**	6	**0.006**	Ekiert & Gogol (1983) [82]	-	0.0833	0.0005 *
Pre- vs. post-treatment	7.04	31.06%	0.218	0	0.444	-	-	0.4694	0.0781
WASO	Q	I^2^	*p*-value	Fail-safe N	*p*-value	Outlier	Overly influential	Rank correlation (*p*-value)	Regression test (*p*-value)
Drug-free BD (depressed) vs. HC	37.39	77.44%	**<0.001**	26	**<0.001**	Feinberg et al. (1982) [55]	-	0.3988	0.4175
Drug-free BD (manic) vs. HC	2.46	0.00%	0.483	1	**0.04**	-	-	0.75	0.6811
Drug-free BD (euthymic) vs. HC	0.203	0.00%	0.904	0	0.18	-	-	1	0.8948
Drug-free BD (depressed) vs. UD	49.813	81.75%	**<0.001**	20	**0.003**	Fossion et al. (1998) [75]	-	0.1646	0.5607
Treated BD vs. HC	16.829	73.04%	**0.002**	8	**0.005**	Leveille et al. (2025) [93]	-	0.0833	<0.0001 *
Pre- vs. post-treatment	9.145	31.44%	0.166	0	0.248	-	-	1	0.6502
ST1	Q	I^2^	*p*-value	Fail-safe N	*p*-value	Outlier	Overly influential	Rank correlation (*p*-value)	Regression test (*p*-value)
Drug-free BD (depressed) vs. HC	12.201	62.12%	**0.016**	0	0.337	Thase et al. (1989) [57]	Thase et al. (1989) [57]	0.8167	0.0023 *
Drug-free BD (manic) vs. HC	4.961	35.26%	0.175	4	**0.014**	-	-	0.3333	0.0565
Drug-free BD (euthymic) vs. HC	7.482	86.64%	**0.006**	0	0.3627	-	-	1	-
Drug-free BD (depressed) vs. UD	2.02	0.00%	0.98	0	0.126	-	Kupfer et al. (1974) [73]	0.7614	0.4473
Treated BD vs. HC	2.22	0.00%	0.818	3	**0.025**	-	-	0.2722	0.2796
Pre- vs. post-treatment	3.469	0.00%	0.748	0	0.09	Maggini et al. (1974) [79]	Maggini et al. (1974) [79]	0.069	0.8125
%ST1	Q	I^2^	*p*-value	Fail-safe N	*p*-value	Outlier	Overly influential	Rank correlation (*p*-value)	Regression test (*p*-value)
Drug-free BD (depressed) vs. HC	21.255	76.22%	**<0.001**	0	0.205	Thase et al. (1989) [57]	Thase et al. (1989) [57]	0.2333	0.0001 *
Drug-free BD (manic) vs. HC	18.44	76.90%	**0.001**	0	0.181	-	-	0–4833	0.2051
Drug-free BD (euthymic) vs. HC	7.045	85.81%	**0.008**	0	0.495	-	-	1	-
Drug-free BD (depressed) vs. UD	9.413	10.98%	0.309	0	0.076	-	-	0.1194	0.0789
Treated BD vs. HC	3.022	0.00%	0.806	9	**0.007**	-	Leveille et al. (2025) [93]	0.5619	0.2082
Pre- vs. post-treatment	2.535	0.00%	0.865	0	0.218	Jindal et al. (2003) [83]	Jindal et al. (2003) [83]	0.0028 *	0.0014 *
ST2	Q	I^2^	*p*-value	Fail-safe N	*p*-value	Outlier	Overly influential	Rank correlation (*p*-value)	Regression test (*p*-value)
Drug-free BD (depressed) vs. HC	17.947	77.41%	**0.001**	3	**0.023**	-	-	0.2333	0.0531
Drug-free BD (manic) vs. HC	1.004	0.00%	0.8	10	**0.001**	-	-	1	0.9388
Drug-free BD (euthymic) vs. HC	0.095	0.00%	0.758	0	0.0849	-	-	1	-
Drug-free BD (depressed) vs. UD	23.912	67.36%	**0.002**	9	**0.012**	-	-	0.3585	0.9517
Treated BD vs. HC	2.756	0.00%	0.738	0	0.221				
Pre- vs. post-treatment	8.057	0.00%	0.234	0	0.175	-	-	0.7726	0.851
%ST2	Q	I^2^	*p*-value	Fail-safe N	*p*-value	Outlier	Overly influential	Rank correlation (*p*-value)	Regression test (*p*-value)
Drug-free BD (depressed) vs. HC	10.632	62.29%	**0.031**	0	0.259	-	-	0.4833	0.3786
Drug-free BD (manic) vs. HC	34.03	86.94%	**<0.001**	4	**0.014**	Asaad et al. (2016) [63]	-	0.4833	0.8518
Drug-free BD (euthymic) vs. HC	1.67	40.13%	0.196	0	0.059	-	-	1	-
Drug-free BD (depressed) vs. UD	12.96	38.76%	0.113	0	0.239	-	-	1	0.9594
Treated BD vs. HC	5.248	13.86%	0.512	0	0.332	-	-	0.7726	0.1737
Pre- vs. post-treatment	8.588	31.67%	0.198	0	0.118	-	-	0.3813	0.0398 *
DELTA	Q	I^2^	*p*-value	Fail-safe N	*p*-value	Outlier	Overly influential	Rank correlation (*p*-value)	Regression test (*p*-value)
Drug-free BD (depressed) vs. HC	5.497	1.14%	0.600	20	**0.001**	-	-	0.5484	0.4931
Drug-free BD (manic) vs. HC	2.271	0.00%	0.518	0	0.487	-	-	0.75	0.4345
Drug-free BD (euthymic) vs. HC	0.577	0.00%	0.749	0	0.407	-	-	0.3333	0.5001
Drug-free BD (depressed) vs. UD	93.952	82.20%	**<0.001**	39	**<0.001**	-	-	0.6265	0.1258
Treated BD vs. HC	10.224	54.00%	0.069	0	0.429	-	-	0.4694	0.2089
Pre- vs. post-treatment	22.747	78.28%	**<0.001**	28	**<0.001**	Kupfer et al. (1974) [73]	.	0.2389	0.0060 *
%DELTA	Q	I^2^	*p*-value	Fail-safe N	*p*-value	Outlier	Overly influential	Rank correlation (*p*-value)	Regression test (*p*-value)
Drug-free BD (depressed) vs. HC	6.124	9.23%	0.525	15	**0.003**	-	-	0.7195	0.8928
Drug-free BD (manic) vs. HC	5.109	29.77%	0.276	0	0.21	-	-	0.8167	0.795
Drug-free BD (euthymic) vs. HC	1.016	0.00%	0.602	0	0.465	-	-	0.3333	0.4132
Drug-free BD (depressed) vs. UD	69.212	80.25%	**<0.001**	6	**0.028**	-	-	0.9226	0.3738
Treated BD vs. HC	6.102	0.00%	0.412	0	0.295	-	-	0.2389	0.1365
Pre- vs. post-treatment	20.39	74.73%	**0.005**	0	0.083	Kupfer et al. (1974) [73]	-	0.3988	0.0316 *
REMT	Q	I^2^	*p*-value	Fail-safe N	*p*-value	Outlier	Overly influential	Rank correlation (*p*-value)	Regression test (*p*-value)
Drug-free BD (depressed) vs. HC	32.689	73.40%	**<0.001**	0	0.238	-	-	1	0.6818
Drug-free BD (manic) vs. HC	0.633	0.00%	0.889	6	**0.005**	-	-	0.3333	0.5762
Drug-free BD (euthymic) vs. HC	1.771	0.57%	0.413	2	**0.022**	-	-	1	0.7268
Drug-free BD (depressed) vs. UD	35.97	62.71%	**<0.001**	2	**0.041**	Duncan et al. (1979) [54]	-	0.1289	0.0045 *
Treated BD vs. HC	12.234	57.65%	**0.032**	14	**0.001**	Soehner et al. (2017) [92]	-	0.4694	0.9055
Pre- vs. post-treatment	33.69	83.67%	**<0.001**	21	**<0.001**	-	-	1	0.4055
%REM	Q	I^2^	*p*-value	Fail-safe N	*p*-value	Outlier	Overly influential	Rank correlation (*p*-value)	Regression test (*p*-value)
Drug-free BD (depressed) vs. HC	14.793	47.45%	0.063	10	**0.009**	-	-	0.2595	0.3198
Drug-free BD (manic) vs. HC	3.328	0.00%	0.504	0	0.337	-	-	0.4833	0.5047
Drug-free BD (euthymic) vs. HC	1.817	0.00%	0.403	11	**<0.001**	-	-	1	0.9550
Drug-free BD (depressed) vs. UD	9.368	0.00%	1	4	**0.03**	-	-	0.1000	0.6315
Treated BD vs. HC	6.921	36.38%	0.227	15	**0.001**	-	-	0.4694	0.7645
Pre- vs. post-treatment	30.511	82.74%	**<0.001**	27	**<0.001**	-	-	1	0.2377
REML	Q	I^2^	*p*-value	Fail-safe N	*p*-value	Outlier	Overly influential	Rank correlation (*p*-value)	Regression test (*p*-value)
Drug-free BD (depressed) vs. HC	110.988	94.56%	**<0.001**	113	**<0.001**	Duncan et al. (1979) [54]	Duncan et al. (1979) [54]	0.6754	0.2205
Drug-free BD (manic) vs. HC	11.733	65.52%	**0.019**	74	**<0.001**	-	-	0.2333	0.2575
Drug-free BD (euthymic) vs. HC	0.641	0.00%	0.726	0	0.151	-	-	1	0.7892
Drug-free BD (depressed) vs. UD	68.333	73.78%	**<0.001**	0	0.433	Duncan et al. (1979) [54]	-	0.1863	0.0315 *
Treated BD vs. HC	1.59	0.00%	0.662	3	**0.019**	-	-	0.7500	0.4133
Pre- vs. post-treatment	22.583	67.04%	**0.002**	88	**<0.001**	-	-	0.7195	0.8350
REMD	Q	I^2^	*p*-value	Fail-safe N	*p*-value	Outlier	Overly influential	Rank correlation (*p*-value)	Regression Test (*p*-value)
Drug-free BD (depressed) vs. HC	57.321	84.47%	**<0.001**	54	**<0.001**	Duncan et al. (1979) [54]	-	1	0.9472
Drug-free BD (manic) vs. HC	6.598	53.54%	0.086	9	**0.002**	-	-	0.7500	0.5173
Drug-free BD (euthymic) vs. HC	0.345	0.00%	0.556	1	0.049	-	-	1	1
Drug-free BD (depressed) vs. UD	16.296	35.52%	0.061	78	**<0.001**	-	-	0.2912	0.0318 *
Treated BD vs. HC	0.102	0.00%	0.95	16	**<0.001**	-	-	1	0.8004
Pre- vs. post-treatment	6.211	30.13%	0.184	0	0.326	-	-	0.8167	0.6750

Statistically significant *p*-value in bold. * Asymmetry found.

## Data Availability

No new data were created or analyzed in this study. Data sharing is not applicable to this article.

## Supplementary Materials

The following supporting information can be downloaded at: https://www.mdpi.com/article/10.3390/brainsci16070742/s1, File S1: PRISMA checklist; File S2: Extended Data.

## Abbreviations

The following abbreviations are used in this manuscript:

BD	Bipolar disorder
DELTA	Delta sleep time
%DELTA	Delta sleep percentage
HC	Healthy controls
NREM	Non-rapid eye movement
PRISMA	Preferred reporting items for systematic reviews and meta-analyses
PSG	Polysomnography
REM	Rapid eye movement
REMD	Rapid eye movement density
REML	Rapid eye movement latency
REMT	Rapid eye movement time
%REM	Rapid eye movement percentage
SD	Standard deviation
SE	Sleep efficiency
SOL	Sleep onset latency
ST1	Stage 1 sleep time
ST2	Stage 2 sleep time
%ST1	Stage 1 sleep percentage
%ST2	Stage 2 sleep percentage
SWS	Slow-wave sleep
TST	Total sleep time
UD	Unipolar depression
WASO	Wake after sleep onset
WoS	Web of Science
